# The “Shiny and Thick High Heel Sign”: A novel radiologic sign for detecting intracranial dural arteriovenous fistulas with time-of-flight magnetic resonance angiography

**DOI:** 10.1007/s00062-021-01036-y

**Published:** 2021-06-08

**Authors:** Hannes Schacht, Inke Regina König, Johannes Hensler, Peter Schramm, Jan Küchler, Claudia Ditz, Alexander Neumann

**Affiliations:** 1 Department of Neuroradiology, University Medical Center Schleswig-Holstein, Lübeck University, Lübeck, Germany; 2 Institute of Medical Biometry and Statistics, University of Lübeck, Lübeck, Germany; 3 Department of Radiology and Neuroradiology, University Medical Center Schleswig-Holstein, Kiel University, Kiel, Germany; 4 Department of Neurosurgery, Lübeck University, Lübeck, Germany

**Keywords:** Middle meningeal artery, Central nervous system vascular disorders, Neurovascular imaging, Pulsatile tinnitus, Radiologic sign

## Abstract

**Purpose:**

Together with the foramen ovale, the middle meningeal artery (MMA) looks like a high heel shoe print on axial time-of-flight magnetic resonance angiography (TOF-MRA) images, with the MMA resembling the heel. Cranial dural arteriovenous fistulas (DAVF) are often fed by the MMA, which can lead to an increase of signal intensity and diameter of this vessel, resulting in a more “shiny” and “thick” high heel print appearance than on the contralateral side. We describe this finding as a novel radiologic sign and provide cut-off values for the ratios of MMA signal intensities and diameters for predicting the presence of a DAVF.

**Methods:**

A total of 84 TOF-MRA examinations of 44 patients with DAVFs (40 with unilateral MMA feeders, 4 with bilateral feeders) and of 40 patients without DAVFs were included. Diameters and signal intensities of both MMAs were measured by two raters and evaluated using receiver operating characteristic analysis.

**Results:**

The diameters of feeding and non-feeding MMAs differed significantly, as did the ratios of signal intensities and of diameters of DAVF and control patients (*P* < 0.0001). Cut-off values were 1.25 for average signal intensity ratio (shiny high heel sign) and 1.21 for diameter ratio (thick high heel sign). The combination of the “shiny” and the “thick” high heel sign resulted in the highest sensitivity (92.5%) and positive predictive value (95%).

**Conclusion:**

The described sign seems promising for the detection of DAVFs with noncontrast-enhanced MRI. The TOF-MRA source images should be reviewed with special attention to the MMA.

## Introduction

Magnetic resonance imaging (MRI) plays an important role in the initial imaging of cranial dural arteriovenous fistulas (DAVF). It has improved over the past years thanks to several novel techniques, such as time-resolved contrast-enhanced magnetic resonance angiography and arterial spin labeling [1–4]. In the field of noncontrast MRI, time-of-flight magnetic resonance angiography (TOF-MRA) is a valuable tool for the detection of DAVFs and can show abnormal findings with high sensitivity and specificity [5].

**Fig. 1:**
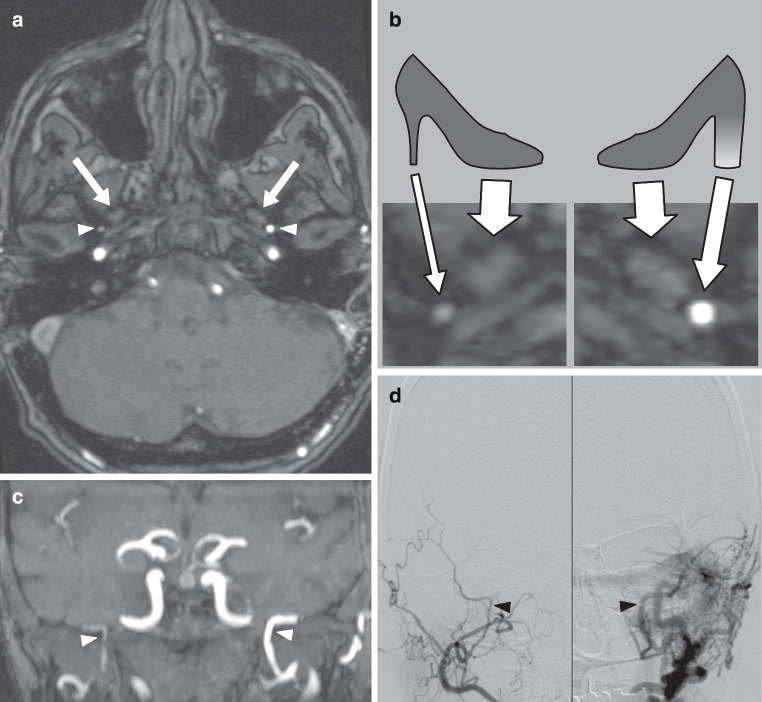
Illustrative case of a 62-year-old woman with a Cognard IIa DAVF on the left side. **a** Axial TOF-MRA image at the level of the foramina spinosa (*arrowheads*) and foramina ovalia (*arrows*) shows increase of diameter and signal intensity of the left MMA. **b** Graphic scheme with enlarged sections from the axial TOF-MRA image demonstrates the high heel sign with a normal, thin high heel print shape on the right side of the patient and a shiny and thick high heel sign on the left side. **c** Coronary TOF-MRA maximum intensity projection (MIP) image shows both MMAs (*arrowheads*) with enlargement and marked intensity on the left side. **d** DSA images show both MMAs (*arrowheads*) with enlargement on the left side and early and retrograde opacification of the left transverse sinus

We aimed to further improve the detection of DAVFs with TOF-MRA by focusing on alterations of the middle meningeal artery (MMA), which is most frequently involved as a feeding vessel and has been reported to be the most common site for transarterial embolization with liquid embolic agents [6, 7]. Together with the foramen ovale, the MMA looks like a high heel shoe print at the level of the foramen spinosum on axial cross-sectional images, with the MMA resembling the “heel”. Normally, there is the appearance of a “stiletto” or thin high heel print. If the MMA feeds a DAVF, it can lead to an increase of diameter and signal intensity (SI) on TOF-MRA images due to higher blood volume and flow. This increase results in a “thick” and/or “shiny” aspect of the high heel print (Fig. 1).

The goal of this case-control study was to describe this phenomenon as a novel radiologic sign and to provide cut-off values for absolute MMA diameter, diameter ratio, and SI ratio for predicting the presence of DAVFs with TOF-MRA.

## Methods

### Patients and Imaging Data

Noncontrast-enhanced axial 3D-TOF-MRA images of 44 consecutive DAVF patients without prior embolization (29 women and 15 men) with a mean age of 61.9 years (range 30–85 years). They were imaged from July 2007 to March 2019 at our institution and retrospectively evaluated by two neuroradiologists, blinded concerning diagnosis and patient histories. The TOF-MRA images were acquired at 1.5 T (*n* = 35) or 3 T (*n* = 9). The diagnosis of DAVF was confirmed by digital subtraction angiography (DSA) in all cases. The control group consisted of 40 patients (28 women and 12 men) with a mean age 60.5 years (range 38–82 years); TOF-MRA images were acquired at 1.5 T (*n* = 29) or 3 T (*n* = 11) without evidence of DAVF on catheter-based panangiography and without evidence of other dural-based lesions. Based on DSA findings, DAVFs were graded using the Cognard classification [8] or, in cases of carotid-cavernous fistulas (CCF), using the Barrow classification [9].

### Measurements

For standardized evaluation, all axial TOF-MRA images were primarily aligned parallel to the anterior commissure-posterior commissure line and only adjusted differently if needed in case of asymmetric skull base anatomy. Measurements were performed using the software IMPAX EE R20 Version XVIII (Agfa HealthCare N.V., Mortsel, Belgium). At the level of the foramina spinosa and foramina ovalia, the MMA diameter and SI were measured on both sides and the diameter ratios and SI ratios were calculated (higher value divided by lower value). Patients with bilateral MMA feeding were included only for analysis of the absolute MMA diameter. Average and maximum SI of the MMA were obtained using a circular region of interest (ROI) with a diameter of 4.0 mm centered on the MMA.

### Statistical Analysis

Measured MMA diameters and calculated ratios are presented as mean with standard deviation (SD) and minimum and maximum values in parentheses. Diameters of fistula-feeding and non-fistula-feeding MMAs, as well as the ratios of diameters and SIs were compared between DAVF patients and control group using the Mann-Whitney U-test. A *P***-**value of < 0.05 was considered to indicate statistical significance. Interrater agreement was estimated using the method proposed by Bland and Altman [10]. Cut-off values for the absolute diameter of DAVF feeding MMAs, diameter ratio and SI ratio for predicting the presence of a DAVF were determined with receiver operating characteristic (ROC) analysis and are presented with the values of the area under the curve (AUC) and 95% confidence interval (CI). Based on the obtained cut-off values, the positive predictive values (PPV) of absolute MMA diameter, diameter ratio, average SI ratio, and the combination of diameter ratio and average SI ratio were calculated. For the combination of diameter ratio and average SI ratio, the higher of both values was used. Statistical analysis was performed using the software SPSS Statistics, Version 25 (IBM, Armonk, NY, USA) and Excel, Version 14 (Microsoft, Redmond, WA, USA).

## Results

### Dural Arteriovenous Fistula and Patient Characteristics

A total of 40 patients (91%) had DAVFs fed unilaterally by 1 MMA. In 4 patients (9%) the DAVF was fed by both MMAs. These patients were not analyzed concerning diameter ratio and SI ratio, but they were included for the analysis of the absolute MMA diameter. Prevalence for Cognard types were 36% (type I, *n* = 16), 16% (type IIa, *n* = 7), 5% (type IIb, *n* = 2), 11% (type IIa + b, *n* = 5), 7% (type III, *n* = 3), 16% (type IV, *n* = 7) and 2% (type V, *n* = 1). Of the patients three had Barrow type D CCF (7%). The DAVF grades and clinical presentation are shown in Table 1.

**Table 1:** Summary of patient and DAVF characteristics

Total number of DAVF patients, *n* (%)	44 (100)
Mean age, years (SD)	61.9 (13.3)
Female, *n* (%)	29 (65.9)
*DAVF classification, n (%)*	
Cognard I	16 (36.4)
Cognard IIa	7 (15.9)
Cognard IIb	2 (4.5)
Cognard IIa + b	5 (11.4)
Cognard III	3 (6.8)
Cognard IV	7 (15.9)
Cognard V	1 (2.3)
Barrow D	3 (6.8)
*Symptoms, n (%)*	
Pulsatile tinnitus	23 (52.3)
Headache	9 (20.5)
Asymptomatic	6 (13.6)
Visual disturbance	5 (11.4)
Vertigo	5 (11.4)
Intracerebral hemorrhage	4 (9.1)
Ataxia	2 (4.5)
Aphasia	2 (4.5)
Dysarthria	1 (2.3)
Subarachnoid hemorrhage	1 (2.3)
Sudden hearing loss	1 (2.3)
Seizures	1 (2.3)
Pulsatile scalp swelling	1 (2.3)

### Interrater Agreement

**Fig. 2:**
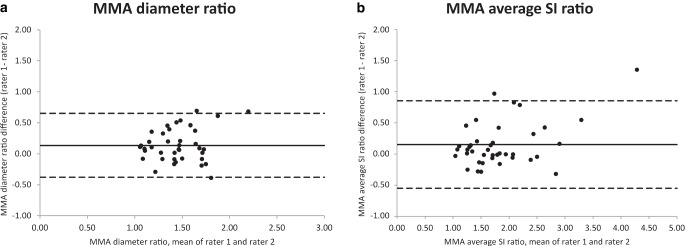
Bland-Altman plots of MMA diameter ratio (**a**) and average SI ratio (**b**)

For the absolute diameter of fistula-feeding MMAs, Bland-Altman analysis showed a mean interrater difference of 0.36 mm with 95% limits of interrater agreement of −0.37 mm to 1.09 mm. For the diameter of non-fistula-feeding MMAs, the mean interrater difference was 0.15 mm (95% limits of agreement −0.42 mm to 0.73 mm). For the MMA diameter ratio, the mean interrater difference was 0.14, with 95% limits of interrater agreement of −0.38 to 0.65. Similar interrater agreement concerning MMA average and maximum SI ratios was noticed, but MMA average SI ratios showed less marked interrater differences in higher ratios. Maximum SI ratios were therefore excluded from further analysis. The Bland-Altman plots of the MMA diameter ratio and average SI ratio are shown in Fig. 2.

### Middle Meningeal Artery Measurements

**Fig. 3:**
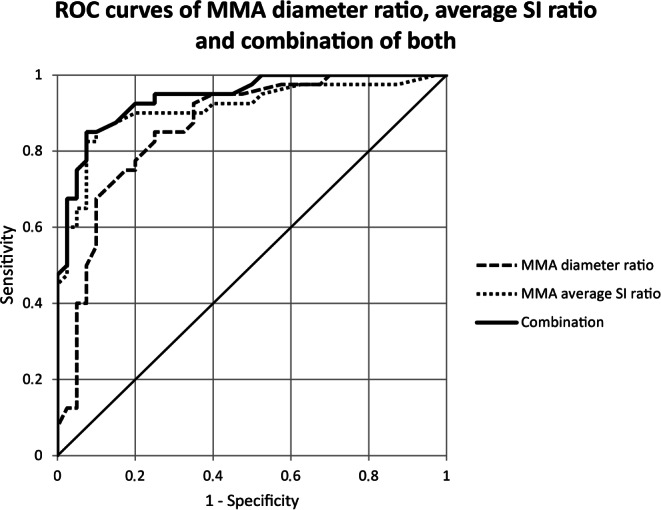
ROC curves of MMA diameter ratio, SI ratio and combination of both

The mean diameter of fistula-feeding MMAs was 2.4 ±0.5 mm (minimum 1.5 mm–maximum 3.3 mm). Non-fistula-feeding MMAs were significantly smaller (*P* < 0.0001) and had a mean diameter of 1.5 ± 0.3 mm (0.8–2.6 mm). In DAVF patients, the mean ratio of MMA diameters was 1.53 ± 0.31 (1.05–2.54). The Mean ratio of MMA average SI was 1.91 ± 0.76 (1.02–4.96). In the control group, mean MMA diameter ratio was 1.16 ± 0.21 (1.00–1.82) and mean MMA average SI ratio was 1.17 ± 0.15 (1.01–1.75). The diameter ratios and the average SI ratios both also differed significantly between DAVF patients and the control group with *P-*values of < 0.0001. The cut-off values were 1.9 mm for the MMA diameter, 1.21 for the MMA diameter ratio, and 1.25 for MMA average SI ratio. Sensitivity and PPV were higher for the MMA average SI ratio (sensitivity 90%, PPV 90%) than for the MMA diameter ratio (sensitivity 85%, PPV 85%) and highest for the combination of both values (sensitivity 92.5%, PPV 95%) (see Fig. 3 and Table 2).

**Table 2:** ROC analysis of MMA diameter, diameter ratio and SI ratio

	AUC (95% CI)	Cut-off value	Sensitivity (%)	Specificity (%)	PPV (%)
1: MMA diameter (mm)	0.91 (0.87–0.96)	1.85	87.5	85.0	87.5
2: MMA diameter ratio	0.86 (0.78–0.95)	1.21	85.0	75.0	85.0
3: MMA average SI ratio	0.91 (0.84–0.98)	1.25	90.0	80.0	90.0
4: Combination of 2 and 3^a^	0.94 (0.89–0.99)	1.25	92.5	80.0	95.0

*AUC* Area under the curve, *CI* Confidence interval, *MMA* Middle meningeal artery, *PPV* Positive predictive value, *ROC* Receiver operating characteristic, *SI* Signal intensity

^a^Maximum of 2 and 3

## Discussion

Digital subtraction angiography remains the gold standard for the diagnostics and treatment of DAVFs; however, MRI is often performed initially when a DAVF is clinically suspected. Time-of-flight MRA can show abnormalities that are highly suggestive of a DAVF and enable gross fistula characterization [11]. Noguchi et al. reported a sensitivity of 100% for high-intensity curvilinear or nodular structures adjacent to sinus walls as well as for high-intensity areas within the venous sinus [5]. Additionally, TOF-MRA has been shown to be useful for documenting complete CCF obliteration after treatment with radiosurgery [12].

Among the many possible DAVF feeding arteries, the MMA is of particular importance, as it is the most common feeding artery and also the most frequently accessed vessel for embolization with Onyx, currently one of the most widely used agents for endovascular treatment of DAVFs [6, 7, 13]. Also, the MMA has been reported to be useful for successful single arterial pedicle embolization of DAVFs, regardless of the type and the number of feeders [14].

The MMA enters the skull base mostly as a branch of the maxillary artery through the foramen spinosum and supplies the biggest part of the cranial dura [15]. It is involved in a variety of diseases besides DAVFs, such as pseudoaneurysms, true aneurysms, moyamoya disease, recurrent chronic subdural hematomas, migraine and meningiomas [16]. Several pathologic processes involving the dura can lead to an enlargement of the MMA. Widening of the foramen spinosum has been described in patients with meningiomas and DAVFs [17, 18]. Takizawa et al. measured the diameters of the anterior MMA branch, distally to the foramen spinosum on TOF-MRA MIP images in patients with chronic subdural hematomas and found significant enlargement on the hematoma side, compared to the non-hematoma side and the control group [19].

In this work, we aimed to improve the sensitivity of TOF-MRA for detecting DAVFs, focusing on MMA alterations due to increased vessel diameter and blood flow. Measuring the exact MMA diameter is limited due to flow velocity-dependent signal intensity and a sometimes blurred depiction of vessel edges on TOF-MRA images [19]. This can be aggravated due to zooming of the source images, which on the other hand is necessary, since the measurements take place in the submillimeter range. Furthermore, local magnetic field inhomogeneity artifacts related to the surrounding bone of the skull base might also affect the measurements. Despite these circumstances, we observed a high interrater agreement regarding the measurement of the absolute MMA diameter, so this parameter appears useful for gross orientation at least and might be especially helpful in cases of bilaterally feeding MMAs, which limit the value of the MMA diameter ratios; however, assuming a constant intrarater measurement inaccuracy when measuring both MMAs, the ratios of the MMA diameters are probably more reliable than the absolute diameters, especially since DAVFs that are fed bilaterally by both MMAs seem to be relatively rare. Also, using the ratios of the MMA measurements should compensate potential inaccuracies related to artifacts due to the surrounding bone. Using equally sized ROIs on both sides enables determination of the MMA signal intensity ratios, which in our results had a higher sensitivity than the diameter ratios. We combined the MMA signal intensity ratio and the diameter ratio, taking into account the higher of both values, which resulted in a high sensitivity and positive predictive value. Our study underlines the importance of reviewing the TOF-MRA source images, as they contain more detailed anatomical information than MIP images, in which small or slow-flowing arteries can be lost [11, 20].

One limitation of this study is its retrospective design. Also, the radiologic sign described in this work cannot serve to rule out a DAVF because there may be several other feeding vessels besides the MMA. Nonetheless, this sign offers a helpful diagnostic tool for scrutinizing TOF-MRA source images for the presence of DAVFs, since the MMA is very often involved as a feeding vessel. The described sign is not specifically related to DAVFs, as there are other dural-based lesions that can alter MMA blood flow and diameter. Further studies in these fields might be of interest since radiological MMA changes might assist in treatment planning.

## Conclusion

We define a positive shiny high heel sign as an MMA average signal intensity ratio of ≥ 1.25 and a positive thick high heel sign as an MMA diameter ratio of ≥ 1.21. Both signs strongly suggest the presence of a DAVF, while the shiny high heel sign is more sensitive than the thick high heel sign, and the combination of both (i.e. if at least one of both signs is positive) provides the highest sensitivity and positive predictive value. Although measurement of the absolute MMA diameter on TOF-MRA images is technically limited, an MMA diameter of 2 mm or more should raise the suspicion of a DAVF. Time-of-flight MRA source images should be reviewed with special attention to the MMAs.
